# Positive feedback loops exacerbate the influence of superspreaders in disease transmission

**DOI:** 10.1016/j.isci.2023.106618

**Published:** 2023-04-11

**Authors:** Klara M. Wanelik, Mike Begon, Andy Fenton, Rachel A. Norman, Pablo M. Beldomenico

**Affiliations:** 1 Department of Evolution, Ecology and Behaviour, Institute of Infection, Veterinary and Ecological Sciences, University of Liverpool, Liverpool, UK; 2 Department of Biology, University of Oxford, Oxford, UK; 3 Department of Computing Science and Mathematics, Faculty of Natural Sciences, University of Stirling, Stirling, UK; 4 Laboratorio de Ecología de Enfermedades, Instituto de Ciencias Veterinarias del Litoral (Consejo de Investigaciones Científicas y Técnicas - Universidad Nacional del Litoral), Esperanza, Argentina

**Keywords:** Health sciences, Medicine, Virology

## Abstract

Superspreaders are recognized as being important drivers of disease spread. However, models to date have assumed random occurrence of superspreaders, irrespective of whom they were infected by. Evidence suggests though that those individuals infected by superspreaders may be more likely to become superspreaders themselves. Here, we begin to explore, theoretically, the effects of such a positive feedback loop on (1) the final epidemic size, (2) the herd immunity threshold, (3) the basic reproduction number, *R*_*0*_, and (4) the peak prevalence of superspreaders, using a generic model for a hypothetical acute viral infection and illustrative parameter values. We show that positive feedback loops can have a profound effect on our chosen epidemic outcomes, even when the transmission advantage of superspreaders is moderate, and despite peak prevalence of superspreaders remaining low. We argue that positive superspreader feedback loops in different infectious diseases, including SARS-CoV-2, should be investigated further, both theoretically and empirically.

## Highlights

• We develop a model where superspreaders are more likely to generate superspreaders
• Results suggest such positive feedback can profoundly affect epidemic outcomes
• Affected outcomes include final epidemic size, herd immunity threshold, and *R*_*0*_

## Graphical abstract

**Figure:**
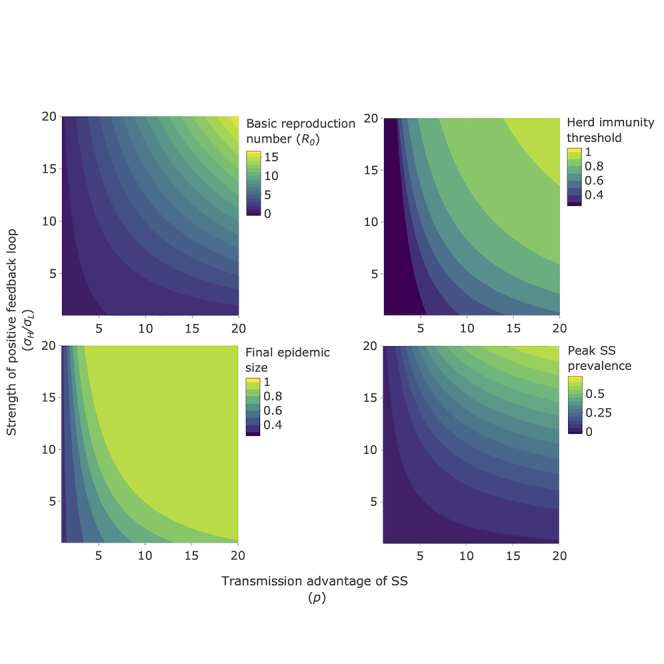

## Introduction

Pathogen transmission requires an appropriate contact between a susceptible and infected individual, such that the susceptible individual is exposed to pathogens shed by the infected individual. Most traditional compartmental models of infectious disease dynamics assume that, on average, all individuals behave in the same way.^1^ However, it has long been known that some individuals, termed “superspreaders”, contribute far more to pathogen transmission, and epidemic spread, than others.^2^^,^^3^^,^^4^^,^^5^ Superspreaders may be defined as individuals in the upper 1% tail of the distribution of pathogen transmission,^6^ but the term has been more widely applied to those with a strongly disproportionate contribution. The importance of accounting for this individual variation in epidemiological models has more recently been recognized, not least because it has played a major role in the ongoing COVID-19 pandemic.^7^^,^^8^^,^^9^^,^^10^^,^^11^^,^^12^^,^^13^

Superspreaders exist for most transmissible diseases of humans, livestock, and wildlife,^14^ and may originate in two ways. First, “supercontactors” transmit infection to more individuals because they have a larger number of contacts than the average, such as in the transmission of HIV and other sexually transmitted diseases (STDs) in humans, through those who are more sexually active,^15^ and of Sin Nombre hantavirus in deer mice.^16^ Second, “supershedders” shed more infectious particles than average and so increase the probability of infection once an appropriate contact has been made, such as when humans co-infected with other STDs shed more HIV,^17^ cattle shed *Escherichia coli* O157 heterogeneously,^18^ or humans have heterogeneous SARS-CoV-2 viral loads.^19^ Previous work has shown that model predictions accounting for superspreaders are very different from average-based approaches, with a higher probability of disease extinction and rarer but more explosive disease outbreaks.^6^ However, for a closed, fixed-sized population, the formula for the final epidemic size is unchanged by the presence of superspreaders.^20^

Multiple studies have explored such heterogeneities in susceptibility and/or contacts and their consequences for disease transmission.^21^^,^^22^^,^^23^^,^^24^^,^^25^ Standard models assume that superspreaders occur irrespective of who they were infected by,^20^ but there is evidence from some systems of positive feedback, recently proposed as an explanation for the heterogeneous propagation pattern of COVID-19,^26^^,^^27^ whereby individuals infected by superspreaders are more likely to be superspreaders themselves. For example, supershedders could generate further supershedders if a higher inoculum dose is more likely to overwhelm the mechanisms of resistance, resulting in poor control of viral replication, causing higher viral loads. This hypothesis has been supported by evidence from experiments in other systems^28^^,^^29^^,^^30^^,^^31^ and, for SARS-CoV-2, from: infections arising from exposure to high doses being more likely to be symptomatic and to have higher intensity^32^^,^^33^; from cases arising from asymptomatic cases being more likely to be asymptomatic^34^^,^^35^; from viral loads driving the size and duration of COVID-19 clusters^36^^,^^37^^,^^38^ and SARS-CoV-2 transmission^39^; and from the ratio of observed-to-expected superspreader-superspreader dyads being greater than expected by chance.^40^

Despite this recognition that superspreaders may generate superspreaders for diseases like COVID-19, no previous study has modeled such positive feedback loops, and hence the role they play in driving the epidemiology of such diseases remains unknown. In this study, therefore, we develop a generic model (for a hypothetical acute viral infection, like SARS-CoV-2 or MERS-CoV) to begin to explore, theoretically, how positive feedback loops affect (1) the final epidemic size, (2) the herd immunity threshold, (3) the basic reproduction number, *R*_*0*_, and (4) the peak prevalence of superspreaders, of an emerging epidemic. By doing so, we hope to stimulate further work, both theoretical and empirical, on this potentially important phenomenon.

## Results

### Modeling positive superspreader feedback loops

To understand the role of the positive feedback loop between superspreaders, we developed a generic model for a hypothetical acute viral infection. The rationale underlying the formulation of this model is based on the hypothesis (detailed above) that supershedders could generate further supershedders. The model includes four classes of hosts: susceptible hosts, infected hosts with low-titre infections (non-superspreaders), infected hosts with high-titre infections (superspreaders; with a transmission advantage over non-superspreaders of magnitude p), and recovered hosts.

The model includes two routes for the generation of superspreaders. It assumes that some proportion of infections ($\sigma_{L}$) from a non-superspreader result in a superspreader—representing the background, spontaneous generation of superspreaders. There are multiple mechanisms by which this could happen. For example, through prolonged contact between the non-superspreader and the recipient, or through the recipient being more susceptible to infection (due to e.g. genetics or an underlying medical conditions).^41^ The model also assumes that some proportion of infections ($\sigma_{H}$) from a superspreader result in another superspreader. Both proportions ($\sigma_{H}$ and $\sigma_{L}$) vary between 0 and 1, but if $\sigma_{H}>\sigma_{L}$, then superspreaders are more likely to generate new superspreaders, and hence, we use $\sigma_{H}/\sigma_{L}$ throughout to represent the strength of the positive feedback loop of superspreader infections.

The full mathematical description of the model and details of its implementation are available in the STAR Methods. The rationale behind the choice of parameter values and initial conditions are also discussed in the STAR Methods.

### Epidemic outcomes reach their highest values when both superspreader advantage and positive superspreader feedback are high

**Figure 1:**
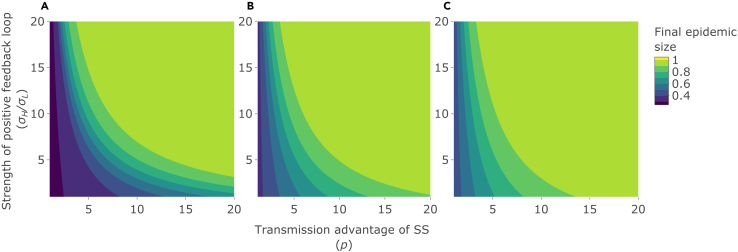
Effects on the final epidemic size Final epidemic size (where the maximum is 1, reflecting everyone in the population becoming infected) as the strength of the positive feedback loop $(\sigma_{H}$/$\sigma_{L}$; y axis), the transmission advantage of superspreaders (SS) (*p*; x axis), and the initial number of SS are varied: (A) initial number of SS = 0% of the total population size. (B) initial number of SS = 5% of the total population size. (C) initial number of SS = 10% of the total population size. See Table 1 for other parameter values.

**Figure 2:**
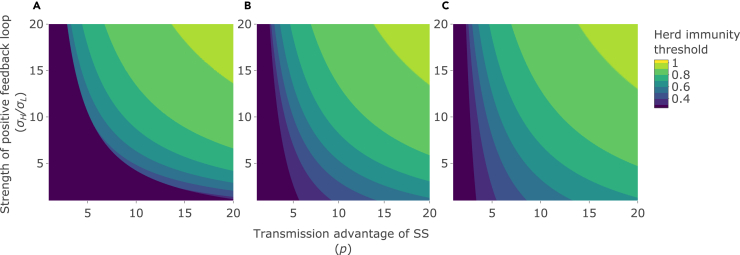
Effects on the herd immunity threshold Herd immunity threshold (the proportion of the population that need to be immune to achieve herd immunity) as the strength of the positive feedback loop $(\sigma_{H}$/$\sigma_{L}$; y axis), the transmission advantage of superspreaders (SS) (*p*; x axis), and the initial number of superspreaders are varied: (A) initial number of SS = 0% of the total population size. (B) initial number of SS = 5% of the total population size. (C) initial number of SS = 10% of the total population size. See Table 1 for other parameter values.

**Figure 3:**
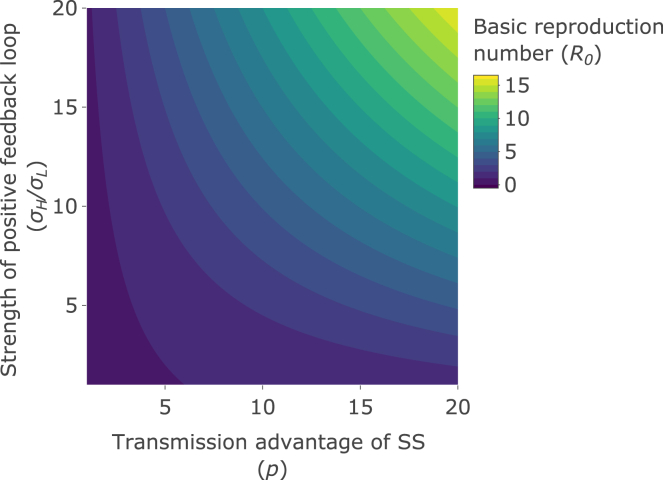
Effects on the basic reproduction number The basic reproduction number (*R*_*0*_) as the strength of the positive feedback loop ($\sigma_{H}/\sigma_{L}$; y axis) and the transmission advantage of superspreaders (SS) (*p*; x axis) are varied. See Table 1 for other parameter values.

The final epidemic size (where the maximum is 1, reflecting everyone in the population becoming infected; Figures 1A–1C), the herd immunity threshold (the proportion of the population that needs to be immune to achieve herd immunity; Figures 2A–2C), and the basic reproduction number, *R*_*0*_ (Figure 3), all increase with increases in the transmission advantage of superspreaders, *p*, and with the strength of the positive feedback loop whereby superspreaders generate further superspreaders, $\sigma_{H}/\sigma_{L}$. Hence, all these epidemic outcomes reach their highest values when both *p* and $\sigma_{H}/\sigma_{L}$ are high.

**Table 1:** Model parameters and their values

Parameter	Symbol	Per capita rate	Comment
Death rate due to disease	α	0	For simplicity, assumed no excess death due to infection.
Recovery rate	γ	0.1	Assumed an average infectious period of one week, as acute viral infections like SARS-CoV-2^38^ and influenza^42^ have infectious periods of approximately one week.
Baseline transmission rate arising from non-superspreaders (*L*-infected hosts)	$\beta_{L}$	8 × 10^−6^	Used an arbitrary $\beta_{L}$ that put the baseline *R*_*0*_ in the absence of superspreading (i.e. when *p* = 1, $\sigma_{H}=\sigma_{L}$) below 1 (see STAR Methods; for the values used here, $R_{0}^{L}=$ 0.8). From this basis, we can explore whether superspreading and/or a positive feedback loop has the potential to drive an epidemic that would not otherwise occur.
Proportion of infections from a non-superspreader (*L*-infected host) that result in a superspreader (*H*-infected host)	$\sigma_{L}$	0.05	Assumed that 5% of infections from a non-superspreader result in a superspreader.
The number of times more superspreaders (*H*-infected hosts) generated by a superspreader (*H*-infected host) than a non-superspreader (*L*-infected host), i.e. the strength of the positive feedback loop	$\sigma_{H}/\sigma_{L}$	1–20	Varied from 1 (equal generation of superspreaders by superspreaders and non-superspreaders) to the maximum feasible value of 20 (20 times more superspreaders generated by a superspreader than a non-superspreader). This maximum feasible value arises since $\sigma_{H}$ cannot exceed 1; given the default value of $\sigma_{L}$ = 0.05, $\sigma_{H}/\sigma_{L}$ cannot exceed 20.
The number of times by which the transmission rate from a superspreader (*H*-infected host) is greater than the transmission rate from a non-superspreader (*L*-infected host; the transmission advantage of superspreaders)	*p*	1–20	Varied from 1 (equal transmission rate from a superspreader and a non-superspreader) to 20 (20 times higher transmission rate from a superspreader than from a non-superspreader). This upper bound is consistent with literature on the superspreading of aerosols for acute viral infections, like SARS-CoV-2^43^

### Positive superspreader feedback loops can have little or no effect on epidemic outcomes in some scenarios

Of course, in the absence of superspreading (*p* = 1), all epidemiological measures are insensitive to $\sigma_{H}/\sigma_{L}$ since there is no superspreader advantage on which a feedback loop might act (along the vertical axes of the figures). Indeed, as shown in STAR Methods, if the pathogen is unable to sustain itself in the absence of superspreading (i.e. if *R*_*0*_ < 1 for a pure low-titre pathogen), there is a lower threshold value of *p*, given by $\frac{\left(\alpha +\gamma \right)}{N\beta_{L}}$, below which no viable amount of positive feedback can drive *R*_*0*_ > 1 (for our baseline parameter values, this lower threshold value of *p* is 1.25, suggesting superspreaders need at least a 25% transmission advantage for an epidemic to occur, regardless of the extent of positive feedback).

### Positive superspreader feedback loops can have a profound effect on epidemic outcomes in other scenarios

Conversely, in the absence of a feedback loop, as assumed in previous studies ($\sigma_{H}/\sigma_{L}$ = 1, along the horizontal axis in the figures), these predicted outcomes, especially *R*_*0*_, may change little with superspreader advantage. For example, in the absence of feedback, and using the baseline parameter values to generate Figure 3, *R*_*0*_ is less than 1 (the pathogen fails to cause an epidemic) unless *p* exceeds 6 (see STAR Methods), and only reaches 1.6 as *p* approaches 20 (the maximum illustrative value used here). But when there is feedback, and especially when the feedback loop is strong (e.g. $\sigma_{H}/\sigma_{L}$ = 20 in Figure 3), *R*_*0*_ rises rapidly, reaching 16 in Figure 3 as *p* approaches 20, and causing an epidemic (*R*_*0*_ > 1) with only very moderate levels of superspreader advantage (*p* ≥ 1.25; see STAR Methods).

### Positive superspreader feedback loops can have a profound effect on epidemic outcomes even when the proportion of superspreaders in the population remains low

**Figure 4:**
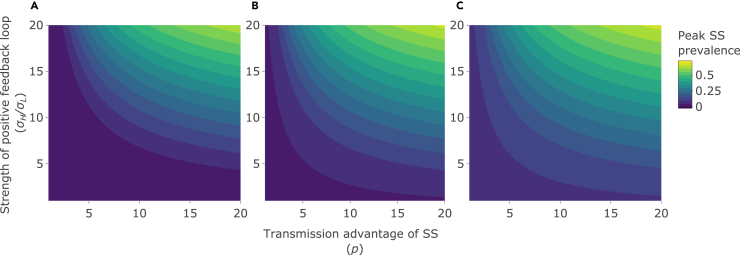
Effects on the peak prevalence of superspreaders Peak prevalence of superspreaders (as a proportion of total population size, *N*) as the strength of the positive feedback loop $(\sigma_{H}$/$\sigma_{L}$; y axis), the transmission advantage of superspreaders (SS) (*p*; x axis), and the initial number of SS are varied: (A) initial number of SS = 0% of the total population size. (B) initial number of SS = 5% of the total population size. (C) initial number of SS = 10% of the total population size. See Table 1 for other parameter values.

It is also noteworthy that this newly identified effect of the feedback loop on our chosen epidemic outcomes can be profound even when the proportion of superspreaders in the population remains low. For example, at intermediate levels of superspreader advantage (p = 5), increasing feedback loop strength from 1 to 5, an intermediate level of feedback, has a powerful effect on both final epidemic size (Figures 1A–1C) and *R*_*0*_ (Figure 3), with *R*_*0*_, for example, increasing from less than 1 (pathogen failing to cause an epidemic), to more than 1 (pathogen causing an epidemic). This is despite the peak prevalence of superspreaders (as a proportion of total population size) not exceeding ∼0.5%–10%, depending on the initial number of superspreaders (Figures 4A–4C).

## Discussion

Superspreaders are recognized as being important drivers of disease spread. However, models to date have assumed random generation of superspreaders, irrespective of whom they were infected by. Here, by contrast, and supported by a range of studies, we explore the importance of positive superspreader feedback loops, whereby those individuals infected by superspreaders are more likely to be superspreaders themselves. We show that superspreaders in their own right (i.e. without positive superspreader feedback), contrary to widespread perception, may have little effect on our chosen epidemic outcomes (namely, the final epidemic size, the herd immunity threshold, and the basic reproduction number, *R*_*0*_), but when positive superspreader feedback is included, the effect may be profound, even when the transmission advantage of superspreaders is moderate, and despite peak prevalence of superspreaders remaining low. Thus, our theoretical framework formally supports and enhances the ideas proposed by Beldomenico,^26^ whereby positive feedback loops have the potential to drive the heterogeneous propagation pattern of a range of infectious diseases, including SARS-CoV-2.

In the context of COVID-19, previous work exploring the effects of population heterogeneity on herd immunity has suggested that assortative mixing, whereby supercontactors tend to preferentially contact other supercontactors, has no effect on the herd immunity threshold.^24^ Furthermore, heterogeneity itself may decrease the threshold, when the proportion of infected (and subsequently immune) individuals in subgroups with the highest contact rates is higher than in subgroups with low contact rates.^25^ In contrast, here, we show that positive feedback loops, whereby supershedders tend to generate more supershedders, *increase* the herd immunity threshold, potentially to a significant degree. This difference reflects supershedding not being a fixed attribute of an individual in our model, such that superspreaders continue to be generated during the course of an epidemic, rather than being among the first to become immune (or die).

Our model is necessarily simple. Our aim was, as a first step, to identify the potential effect of positive superspreader feedback loops on a number of epidemic outcomes. Given the results from this initial study, we contend that the existence of positive feedback loops in different host-pathogen systems warrants further investigation. It will be important to test our model using empirical data, and to further develop our model theoretically, to quantify more precisely the effects of positive feedback loops, and to incorporate further biological details.

### Limitations of the study

We present results (generated entirely via numerical simulation) from a generic model, parameterized using illustrative values to represent a hypothetical acute viral infection, as an initial exploration of this potentially important phenomenon. Unfortunately, we did not have access to sufficient data to parameterize our model, or to test our model, for a specific pathogen. However, we hope that others who do, will adapt and parameterize our model for a specific pathogen and, in so doing, further interrogate our model and its predictions.

## STAR★Methods

### Key resources table

**Table:** 

REAGENT or RESOURCE	SOURCE	IDENTIFIER
**Software and algorithms**		

R version 4.0.3	R Foundation for Statistical Computing	https://www.r-project.org

**Deposited Data**		

R code to perform simulations	This paper	https://github.com/kwanelik/Positive-superspreader-feedback-loops https://doi.org/10.5281/zenodo.7767626

### Resource availability

#### Lead contact

Further information and requests for resources should be directed to and will be fulfilled by the lead contact, Klara M Wanelik (klara.wanelik@biology.ox.ac.uk).

#### Materials availability

This study did not generate new unique reagents.

### Method details

We initially provide a broad overview of the model structure and metrics we used to quantify the impacts of positive superspreader feedback loops on an epidemic; we provide more detailed explanations and calculations relating to those metrics further down in an ‘additional calculations’ section.

#### Mathematical description of the model

To understand the role of the positive feedback loop between superspreaders we developed the following model:

(Equation 1) $$\frac{dS}{dt}=-S\beta_{L}\left(L+pH\right)\text{,}$$

(Equation 2) $$\frac{dL}{dt}=S\beta_{L}\left(\left(1-\sigma_{L})L+p(1-\sigma_{H}\right)H\right)-\left(\alpha +\gamma \right)L\text{,}$$

(Equation 3) $$\frac{dH}{dt}=S\beta_{L}\left(\sigma_{L}L+\sigma_{H}pH\right)-\left(\alpha +\gamma \right)H\text{,}$$

(Equation 4) $$\frac{dR}{dt}=\gamma \left(H+L\right)\text{.}$$

where *S* refers to the number of susceptible hosts, *L* to infected hosts with low-titre infections (non-superspreaders), *H* to infected hosts with high-titre infections (superspreaders) and *R* to recovered hosts. $\beta_{L}$ represents the baseline transmission rate arising from *L*-infected hosts. This baseline rate is then increased through transmission by superspreaders (*H*-infected hosts) by a magnitude p (we assume p≥1) representing the factor by which the transmission rate from a superspreader is greater than the transmission rate from a non-superspreader (i.e. the transmission advantage of superspreaders); Mathematically, p has no upper bound.

$\sigma_{L}$ is the proportion of infections from a non-superspreader that result in a superspreader, and so represents the background, spontaneous generation of superspreaders. $\sigma_{H}$ is the proportion of infections from a superspreader that result in another superspreader. $\sigma_{H}$ and $\sigma_{L}$ both vary between 0 and 1, but if $\sigma_{H}>\sigma_{L}$ then superspreaders are more likely to generate new superspreaders, and hence we use $\sigma_{H}/\sigma_{L}$ to represent the strength of the positive feedback loop of superspreader infections.

*α* is the death rate due to disease, and *γ* the recovery rate from the disease (which may be through natural recovery, or recovery through hospitalisation), both assumed to be the same for superspreaders and non-superspreaders. For this model, there are no natural births or deaths, so the population only changes in size due to deaths resulting from the disease. So, if *α* = 0 then the population remains constant, size *N* = *S* + *L* + *H* + *R*.

#### Calculating epidemic outcomes

For an infection spreading through a closed, fixed-sized population, it is possible to calculate the final epidemic size – the total number of individuals infected throughout the epidemic.^44^ Ma and Earn^20^ showed that the formula for the final epidemic size, *Z*, is unchanged by the presence of a fixed proportion of superspreaders:

(Equation 5) $$Z=S\left[0\right]\left(1-\mathrm{Exp}\left(-Z\frac{\beta_{L}}{\alpha +\gamma }\left(1-\sigma_{H}+p\sigma_{L}\right)-\left(\frac{\beta_{L}}{\gamma +\alpha }\right)\left(L\left[0\right]+p\;H\left[0\right]\right)\right)\right)\text{.}$$

Rescaling to express the sizes of each class as proportions of a fixed population size of 1, and in the limit I(0)→0,S(0)→1 the final epidemic size, *Z*, for all of the models they considered, is given by $Z=1-e^{-R_{0}Z}$.^20^

For the current model, when $\sigma_{H}=\sigma_{L}$ (i.e. when there is no positive feedback loop) the final epidemic size, *Z*, is given by the same formula (see ‘additional calculations’ section below). However, when $\sigma_{H}>\sigma_{L}$, and so the positive feedback loop does exist, it is not possible to use the methods of Ma and Earn^20^ to calculate the final epidemic size, since additional non-linearities in this model disqualify the simplifications they use. However, in the ‘additional calculations’ section below we show that when $\sigma_{H}>\sigma_{L}$, the final epidemic size will be larger than in the absence of such a feedback loop, and this difference increases as the difference between $\sigma_{H}$ and $\sigma_{L}$ increases. In the Results, we quantify the final epidemic size numerically by running simulations in R version 4.0.3^45^ using the de-Solve package^46^ while varying the ratio $\sigma_{H}/\sigma_{L}$ (the strength of the positive feedback loop, assuming $\sigma_{H}/\sigma_{L}\geq 1$) and *p* (the transmission advantage of superspreaders, assuming *p* ≥ 1). We also calculate numerically the peak prevalence of superspreaders and the herd immunity threshold i.e. the proportion of the population that needs to be immune to achieve herd immunity. The latter is calculated by subtracting from one the proportion of the population that remains susceptible when the number of infecteds peaks.^23^ Finally, we calculate the basic reproduction number (*R*_*0*_), i.e. the number of new infections generated by one infectious individual in a completely susceptible population for each of these scenarios. Using the next generation method of Diekmann et al.^47^ (see ‘additional calculations’ section below), *R*_*0*_ for this model is given by:

(Equation 6) $$R_{0}=\frac{N\beta_{L}\left(1+p\sigma_{H}-\sigma_{L}+\sqrt{{\left(\sigma_{L}-1-p\sigma_{H}\right)}^{2}-4p\left(\sigma_{H}-\sigma_{L}\right)}\right)}{2\left(\alpha +\gamma \right)}\text{.}$$

#### Choice of parameter values and initial conditions

Parameter values are chosen to represent a hypothetical acute viral infection for illustrative purposes, and are consistent with the literature on acute viral infections like SARS-CoV-2, MERS-CoV and influenza (see Table 1). Again, for illustrative purposes, the initial number of non-superspreader infecteds, *L*, was kept constant at 10% of the total population size, *N*, as was the initial number of recovereds, *R*, at 0%. We varied the initial number of superspreaders, *H*, between 0–10% (consistent with previous modelling of acute viral infections, like MERS-CoV^48^). The total population size, *N*, was set to 10,000 – the population size of a small town in the UK. All models were run for sufficient periods of time to ensure that the epidemic had completed in all cases.

#### Additional calculations

From Equation (1) dividing both sides by S and integrating both sides with respect to t

(Equation 7) $$\log\left[\frac{S\left(\infty \right)}{S\left(0\right)}\right]=-\beta_{L}\int_{0}^{\infty }L\;dt-p\beta_{L}\int_{0}^{\infty }H\;dt\text{.}$$

To solve Equation (2) we rearrange Equation (1) to get $\beta_{L}pSH$ in terms of S and L and substitute into Equation (2). Integrating both sides with respect to t and assuming that L(∞)=0 gives:

(Equation 8) $$-L\left(0\right)=\left(\sigma_{H}-1\right)\left[S\left(\infty \right)-S\left(0\right)\right]+\int_{0}^{\infty }S\beta_{L}L\left(\sigma_{H}-\sigma_{L}\right)\;dt-\left(\gamma +\alpha \right)\int_{0}^{\infty }L\;dt\text{.}$$

To solve Equation (3) we rearrange Equation (1) to get $\beta_{L}SL$ in terms of S and H and substitute into Equation (3). Integrating both sides with respect to t and assuming that H(∞)=0 gives:

(Equation 9) $$-H\left(0\right)=-\sigma_{L}\left[S\left(\infty \right)-S\left(0\right)\right]+\int_{0}^{\infty }S\beta_{L}pH\left(\sigma_{H}-\sigma_{L}\right)\;dt-\left(\gamma +\alpha \right)\int_{0}^{\infty }H\;dt\text{.}$$

The final epidemic size is the difference between the number of susceptibles at t=0 and t=∞ i.e. Z=S(0)−S(∞). Rearranging Equations (8) and (9) in order to get the integrals of H and L with respect to time, and substituting into Equation (7) gives:

(Equation 10) $$\log\left[\frac{S\left(\infty \right)}{S\left(0\right)}\right]=-\frac{\beta_{L}}{\alpha +\gamma }\left(L\left(0\right)+pH\left(0\right)-\left(1-\sigma_{H}+p\sigma_{L}\right)Z+\left(\sigma_{H}-\sigma_{L}\right)\beta_{L}\int_{0}^{\infty }SL+p^{2}SH\;dt\right)\text{.}$$

When $\sigma_{H}=\sigma_{L}$ this gives the same solution as in Ma and Earn,^20^ and for a fixed population size of 1 and in the limit I(0)→0,S(0)→1 the final epidemic size, Z, for all of the models they considered, is given by $Z=1-e^{-R_{0}Z}$. However, in a model where there is a positive feedback loop, with superspreaders generating more superspreaders, there is an extra positive term which makes the exponent of the exponential term larger and still negative and so makes the final epidemic size bigger; the larger the difference between $\sigma_{H}$ and $\sigma_{L}$, the larger the impact on the final epidemic size.

2. Calculating the basic reproduction number, $R_{0}$, using the method of Diekmann et al.^47^

We calculated $R_{0}$ following the next generation approach of Diekmann et al.,^47^ where $R_{0}$ is the dominant eigenvalue of the matrix $K_{L}$, obtained from the transmission matrix T and the inverse of the transition matrix $\Sigma \;\left(K_{L}=-T\Sigma^{-1}\right)$, derived from the equations of the system presented above, and evaluated at the disease-free equilibrium (population of size N, comprising all susceptible individuals):

(Equation 11) $$T=\left(\begin{array}{cc} N\beta_{L}\left(1-\sigma_{L}\right) & pN\beta_{L}\left(1-\sigma_{H}\right)\\ N\beta_{L}\sigma_{L} & pN\beta_{L}\sigma_{H} \end{array}\right)\text{.}$$

(Equation 12) $$\Sigma =\left(\begin{array}{cc} -\left(\alpha +\gamma \right) & 0\\ 0 & -\left(\alpha +\gamma \right) \end{array}\right)\text{.}$$

and so

(Equation 13) $$K_{L}=\left(\begin{array}{cc} \frac{N\beta_{L}\left(1-\sigma_{L}\right)}{\left(\alpha +\gamma \right)} & \frac{pN\beta_{L}\left(1-\sigma_{H}\right)}{\left(\alpha +\gamma \right)}\\ \frac{N\beta_{L}\sigma_{L}}{\left(\alpha +\gamma \right)} & \frac{pN\beta_{L}\sigma_{H}}{\left(\alpha +\gamma \right)} \end{array}\right)\text{.}$$

The dominant eigenvalue ($R_{0}$) of $K_{L}$ is, as presented above:

(Equation 6) $$R_{0}=\frac{N\beta_{L}\left(1+p\sigma_{H}-\sigma_{L}+\sqrt{{\left(\sigma_{L}-1-p\sigma_{H}\right)}^{2}-4p\left(\sigma_{H}-\sigma_{L}\right)}\right)}{2\left(\alpha +\gamma \right)}\text{.}$$

This expression always exists and is non-negative if $1\geq \sigma_{L}$ and $1\geq \sigma_{H}$ (i.e. if superspreaders are at least as likely to generate new superspreaders as non-superspreaders are, as assumed for our model).

We also note that in the absence of superspreading ($p=\sigma_{H}=$ $\sigma_{L}=0$), the basic reproduction number for a pathogen purely transmitting via low-titre, non-superspreaders, is:

(Equation 14) $$R_{0}^{L}=\frac{N\beta_{L}}{\alpha +\gamma }\text{.}$$

3. Threshold superspreading rates for an epidemic

From the above expression for $R_{0}$ we can calculate analytical expressions for key threshold values of p (the relative transmission advantage of superspreading) for an epidemic to take off ($R_{0}\geq 1$):

  (i) How strong does superspreading need to be to allow an epidemic, when transmission from superspreaders always generates new superspreaders (i.e. when $\sigma_{H}=1$)?

Setting $\sigma_{H}=1$ in the above expression for $R_{0}$, setting that expression equal to 1 (the threshold for an epidemic to occur), and solving for p gives:

(Equation 15) $$p_{1}=\frac{\left(\alpha +\gamma \right)}{N\beta_{L}}=\frac{1}{R_{o}^{L}}\text{.}$$

For the baseline parameter values used to generate Figure 3, $p_{1}=1.25$.

Hence, even if superspreaders always generate new superspreaders ($\sigma_{H}=1$), if $R_{o}^{L}<1$ (such that non-superspreaders alone are not able to cause an epidemic) there is a lower limit of p that is greater than 1, that must be exceeded in order for an epidemic to occur. In other words, even 100% transmission of superspreading is not necessarily sufficient to drive an epidemic of a pathogen with only mild levels of superspreading.

  (ii) How strong does superspreading need to be to drive an epidemic, when there is no positive feedback loop between superspreaders (i.e. when $\sigma_{H}=\sigma_{L}$)?

Setting $\sigma_{H}=\sigma_{L}$ in the above expression for $R_{0}$, setting that expression equal to 1 and solving for p gives:

(Equation 16) p2=α+γ−NβL(1−σL)NβLσL=1RoL−(1−σL)σL=p1−(1−σL)σL.

Hence in the absence of a positive feedback loop in superspreader transmission, the rate of superspreader advantage (p) needs to exceed a threshold value determined by the baseline $R_{0}$ in the absence of superspreading ($R_{o}^{L}$) and the rate at which non-superspreaders spontaneously generate superspreaders ($\sigma_{L}$), in order for an epidemic to occur. This threshold is shown in Figure 3 by the value of p along the x-axis (when $\sigma_{H}=\sigma_{L}$), at the boundary where $R_{0}=1$. For the baseline parameter values used to generate Figure 3, $p_{2}=6$.

### Quantification and statistical analysis

Results were generated via numerical simulation in R version 4.0.3.

## Data Availability

• No empirical data were used in this paper. • All original code for numerics and figure production has been deposited on GitHub and is publicly available. DOI is listed in the key resources table. • Any additional information required to reanalyze the data reported in this paper is available from the lead contact upon request.
